# Self-Efficacy and Adherence Behaviors in Rheumatoid Arthritis Patients

**DOI:** 10.5888/pcd15.180218

**Published:** 2018-10-18

**Authors:** Christiana Oshotse, Leah L. Zullig, Hayden B. Bosworth, Pikuei Tu, Cheryl Lin

**Affiliations:** 1 Department of Population Health Sciences, Duke University, Durham, North Carolina; 2 Center for Health Services Research in Primary Care, Durham Veterans Affairs Health Care System, Durham, North Carolina; 3 Departments of Psychiatry and School of Nursing, Duke University, Durham, North Carolina; 4 Duke University Policy and Organizational Management Program, Durham, North Carolina

## Abstract

**Introduction:**

Rheumatoid arthritis (RA) is a common disease that requires patient self-management with chronic medications. Adherence rates for RA medications are suboptimal. This study explores medication adherence and self-efficacy behaviors among RA patients.

**Methods:**

We conducted a qualitative study comprising focus groups and individual interviews. Nineteen participants were recruited and screened to participate in three 90-minute focus groups (n = 13) and six 60-minute individual interviews. We created and maintained a codebook to analyze data. Interviews were analyzed by using NViVo qualitative analysis software.

**Results:**

Key points in participant interviews were 1) self-efficacy as influenced by the ability to establish routines, and having an understanding relationship with their healthcare provider; 2) self-efficacy to adjust medications depended on having permission from providers to adjust medications, perceptions of the effectiveness of medications, and confidence in self-knowledge to make appropriate adjustments; and 3) changes in self-efficacy over time were influenced by initial denial and later acceptance of the diagnosis. Participant interviews revealed that medication adherence is a spectrum that ranges from adherent to nonadherent.

**Conclusion:**

Participants’ experience with RA medications revealed varied underlying reasons for adherence behaviors. Recognizing adherence as a dynamic behavior has important implications for how adherence interventions are designed. For example, participants reported adjusting medications in response to the unpredictable nature of RA. Interventions could collect information about RA symptoms and be tailored to provide adherence support at times when patients need it most. The importance of self-efficacy in influencing participants’ adherence behaviors is an area for continuing research among patients and providers.

## Introduction

Rheumatoid arthritis (RA) patients rely on fast-acting NSAIDs (nonsteroidal anti-inflammatory drugs) to reduce inflammation and slow-acting DMARDs (disease-modifying antirheumatic drugs) to delay disease progression (1). Although overall adherence to chronic disease medications is approximately 50% (2), RA studies report adherence rates ranging from 30% to 80% (3).

Medication adherence is the extent to which patients start and persist with prescribed regimens (2,4). Deviations from this protocol constitute nonadherence (5,6). No standardized guide exists for management of RA pain, which varies between patients (3). In response, some patients adjust dosages of prescribed medications to control pain and flare-ups. Characterization of this behavior is absent from the existing literature (3,7).

Barriers to realizing proper adherence can readily mount and decrease an individual’s perception of their ability to adhere (2,8). Self-efficacy is the individual’s belief in their ability to complete specific tasks. Self-efficacy is associated with health-promoting behaviors, such as improving communication with providers (9), engaging in recommended health behaviors (10–12), and adjusting to illness and treatments (13,14). This article operationalizes self-efficacy as an individual’s belief in their ability to follow prescribed medication regimens to achieve improved health outcomes (15).

Although several RA studies have evaluated the relationship between self-efficacy and various social factors (1,8,16–18), few have considered how self-efficacy affects medication adherence (1,19). The primary objective of this study was to examine the association between self-efficacy and medication-taking behaviors in RA patients. The secondary objective included investigating RA patients’ experience with taking medications, a rare perspective in existing studies.

## Methods

A research company recruited participants who self-identified as RA patients and resided in Durham, North Carolina. Focus group participants received a $75 gift card. Flyers were posted in Durham RA clinics to recruit for individual interviews. Interviewees received a $35 gift card.

Participants were included if aged between 18 and 75 years, diagnosed with RA for at least 6 months, currently taking RA medications, and had seen a medical doctor for RA in the past 18 months. Female participants were excluded if pregnant, breastfeeding, or planning to become pregnant within 1 year. Participants were selected to ensure representation by age, gender, ethnicity, length of RA diagnosis, and self-perceived level of adherence. Focus groups presented opportunities for discussion about adherence and medication-taking behavior. Individual interviews were conducted to probe participants’ responses without group dynamic influence.

Two 90-minute focus groups were held in November and December of 2017, respectively. Later, a third focus group (January 2018) and six 60-minute individual interviews (January and February 2018) were conducted. Focus groups and individual interviews were conducted by using a guide (Table 1) shaped by the Health Belief Model (HBM) (20) and developed by multidisciplinary experts in the fields of marketing, psychology, and health services research.

**Table 1:** Excerpt of Focus Group and Interview Discussion Guide, RA Patients, Durham, North Carolina, November 2017 – January 2018

Relationship with	Example Questions
**… disease**	*How has RA impacted your life?* *Tell me about your last bad day?* *Tell me about your last good day?* *What is the most frustrating aspect of having RA?*
**… others/social support**	*What impact does RA have on your family life? Social life?* *How much do you share your condition with family or others?* *Do you interact with anyone else with RA?*
**…Physician/Rheumatologist**	*How much time do you typically spend during a visit with your doctor?* *What is your relationship with your doctor like?* *How much choice do you feel your doctor gives you in terms of treatment?* *How often do you go to your doctor with ideas/information about treatment adjustments?*
**…medication**	*What is it like to have to take medication every day?* *How confident are you that you are taking all your medication correctly?* *How satisfied are you with your medication?* *If your RA were a person — what would they be like?*
**Conclusion**	*Are there any areas about your experience with RA that we did not cover that you’d like to share?*

Abbreviation: RA, rheumatoid arthritis.

A codebook was developed a priori based on research themes. Research analysts sequentially coded the transcripts through a rotational system of primary, secondary, and tertiary reviewers by using NViVo 10 (QSR International Pty Ltd, Version 10). Duke University Institutional Review Board approved this study (IRB # 2018–0156).

## Results

Three focus groups (n = 13) and 6 individual interviews were conducted between November 2017 and February 2018, for a study total of 19 participants. Participants were aged from 18 to 70 years (average age 45 years [SD = 14.8]). Six participants (30%) were male. The average self-reported impact of RA was 4.25 (SD = 1.25) on a scale of 1 to 7 with 1 = no impact and 7 = extreme impact. Most participants (65%) held or were pursuing a bachelor's degree or higher and most (75%) received health insurance through their employer. Participants’ self-reported adherence levels were measured on a scale of 1 to 5, with the average reported adherence level at 4.35 (SD = 0.72). Table 2 further details participant characteristics. Table 3 lists medications participants were prescribed, but does not represent all medications available to RA patients.

**Table 2:** Interview Participants Sociodemographic and Disease-Related Characteristics (N = 19), RA Patients, Durham, North Carolina, November 2017 – January 2018

Characteristic	Percent^a^	SD (Min, Max)
**Age, mean, y**	45.0	14.8 (18.0, 70.0)
**Sex**		
Male	30.0	–
Female	70.0	–
**Race**		
White	65.0	–
Black	17.5	–
Hispanic	17.5	–
**Education**		
High school diploma	15.0	–
Currently in college or Bachelors’ degree	65.0	–
Graduate degree	20.0	–
**Insurance type**		
Self-purchased	20.0	–
Employer provided	75.0	–
Medicare Part D/Medicaid	5.0	–
**Impact of RA, mean^b^**	4.25	1.25 (2.00, 6.00)
**Self-reported adherence level, mean^c^**	4.35	0.72 (3.00, 5.00)
**Average diagnosis, mean, y**	11.13	6.67 (2.50, 27.00)

Abbreviation: RA, rheumatoid arthritis.

a Data are percentages, unless otherwise noted.

b Self-reported Impact of RA: 1=no impact; 7=extreme impact.

c Self-reported Adherence level: 1=never; 2=not often; 3=sometimes; 4=most of the time; 5=always.

**Table 3:** Frequently Prescribed RA Medications

Category	Medications
**DMARDs**	Plaquenil Azulfidine Arava Methotrexate
**Biologics**	Orencia Humira Actrema Enbrel Simponi
**NSAIDs**	Aspirin Celebrex Cambia Naproxen
**Steroids**	Prednisone Decadron

Abbreviations: Biologics, biologic response modifiers; DMARDs, Disease-modifying anti-rheumatic drugs; NSAIDs, non-steroidal anti-inflammatory drugs; RA, rheumatoid arthritis; Steroids, anti-inflammatory drugs.

Participant responses presented self-efficacy as an outcome influenced by participants’ beliefs in the necessity of medication and in the patient–provider relationship. Two types of self-efficacy were most evident in affecting participants’ adherence: confidence in their ability to manage medication and confidence to make adjustments to medication dosages.

### Medication management

Most participants reported having high self-efficacy to manage RA medication. They expressed feeling competent about the function and dosage of their medications. They described developing routines, such as using weekly pill organizers, leaving pills in familiar places in homes, and always taking medications at regular intervals, which allowed them to integrate medication-taking into their daily lives.

I try to take mine at the same time every day. It’s not a big deal. I just get up in the middle of the night, take it, go back to sleep. (Focus Group [FG] 1, Participant 1)

Some participants reported scheduling reminders through devices and having support from close family members who reminded them to take their medications. These participants reported that, over time, the act of taking their medications became natural, often without the need of explicit reminders to take medications.

I just take the medication in the morning, first thing. I don’t get out of bed. I don’t go to the bathroom. I just take it. (Individual Interview [IDI] #5)

Some participants expressed that having supportive healthcare providers who thoroughly explained their RA medications made them feel more capable to take their medications appropriately.

I felt comfortable with her [rheumatologist] because she was very knowledgeable about the different medication types, and I said I would prefer to start on something that has been out there longer with fewer adverse side effects and so that’s how we got on Humira. (IDI #3)

Participants discussed having dealt with insensitive healthcare providers who did not provide necessary guidance. Participants who expressed lower self-efficacy were unsure of the necessity of their medications. They revealed abruptly discontinuing their medications. They were unclear about side effects and unmotivated to continue taking their medications.

She [rheumatologist] would suggest other medications, and I could never get a real clear answer why she thought I should do that instead, and that was just kind of frustrating for me, and consequently I usually didn’t do that [take the medications]. (FG1, Participant 4)

I’ve had other rheumatologists that have said, “That’s not that bad” about my pain. My [current] doctor wants to treat it and figure out if there’s something else that works. I wish I had her [rheumatologist] from the beginning. I wouldn’t have had some of the deformities that I do. (FG2, Participant 2)

A participant’s perception of a positive relationship with their healthcare provider made them feel more comfortable with following the directions and advice of their providers. Most participants expressed currently having positive, communicative relationships with their healthcare providers, which contributed to their comfort with taking their medications.

### Medication adjustment

About half of participants reported intermittently adjusting their medications. They expressed high self-efficacy to make these adjustments. Interviews revealed that participants often received clearance from physicians to adjust fast-acting steroids and NSAIDs, such as prednisone (Table 3), to manage pain and flare-ups. This clearance from providers made participants more confident to adjust medications as needed during episodes of pain and flare-ups.

‘Cause I’ve been taking it so long, I’m really close to my doctor, he told me to do that if anything happens I can go up [on medication dosage]. (FG1, Participant 1)

It was supposed to be two tablets a day, but he [rheumatologist] said that if I see I’m doing okay, I can go down to a pill and a half. Whereas if I see that I’m starting to ache or feel pain, then go back up. (IDI #6)

Other participants adjusted their medications because they were confident in their ability to self-medicate. They expressed knowing their body and pain tolerance better than their providers, thus making them the decision maker on how to adjust medications during flare-ups.

I know my body well enough that I could say that I need 10 mg of prednisone today or for a couple of days. (FG2, Participant 2)

The remaining individuals adjusted medications that were not working appropriately to control regular pain and inflammation. Perceived failure of their medications contributed to their belief of having leeway to make adjustments. Others expressed confidently making adjustments after consulting external sources (eg, relatives, internet). These participants detailed adjustment to DMARDs and Biologics (Table 3), often without consulting their provider beforehand.

I was taking the prescription the way I was supposed to take it, and I wasn’t getting any relief. I just took one extra one, and then the pain did go away. (FG2, Participant 2)

I've been trying to space it [Humira] out as much as I can in-between injections, and I do that at my own discretion. My rheumatologists have gotten on me. They’re like, “You should take it more often.” Because I’ll have a flare-up, and then I’ll try to push through. (IDI #3)

All participants who adjusted their medications stated that they informed their providers either before or immediately after adjusting their medications.

They initially told me it was fine if I skipped a week, because there was still medication in my system, so I did inform her [rheumatologist] whenever that was going to happen . . . (IDI #2)

Some participants who expressed low self-efficacy to make adjustments to their medication regimen were those who did not receive permission from their providers to adjust their medications. These individuals were strongly against adjusting medications. They believed that doing so without provider supervision placed them at risk for possible side effects.

You don’t know what’s going to happen when you change the doses and if you change the doses you change the medication side-effects too. (FG1, Participant 1)

Participants with comorbid conditions in addition to RA often reported that they did not adjust their medications for fear of possible complications. In addition, other participants had regimens titrated to treat the severity of their RA and did not adjust their medications because of the sensitive nature of their regimens.

I can’t take a lot of different medications. Normally the medicines that the doctor prescribes, that’s what I take, because my liver is in stage 4 cirrhosis. (FG2, Participant 5)

### Change in self-efficacy over time

A few participants said that their belief in their ability to manage RA increased as they understood the cause of their symptoms and the purpose of their medications better. They described once having uncertainties in their ability to manage RA because they initially disagreed with their RA diagnosis. These individuals often delayed initiating medications or would abruptly discontinue treatment. The painful and debilitating nature of RA prompted them eventually to adhere to their medication regimen.

One participant expressed what is characterized as a low perceived susceptibility to RA (ie, denial) when initially diagnosed, and her denial influenced her decision initially not to adhere to her medications. She expressed that her denial was attached to her negative perceptions of being prescribed intense and aggressive medications and in her disappointment at her body’s loss of ability.

You’re stubborn at the beginning like, “I’m not sick, leave me alone.” But then the more you have the disease and the more you’re getting damaged, you’ll take the medication. (FG2, Participant 4)

Most other participants reported almost no changes in their self-efficacy over the course of their disease. These individuals have always felt confident in their ability to manage their medication regimens. Many attributed this to supportive health providers, minor impact of RA, uncomplicated medication regimens, and discipline in adhering to medications.

### Adherence as a spectrum

Most participants reported exhibiting both adherent and nonadherent behaviors. They described having major periods of adherent behaviors with occasional intervals of nonadherent events in between that comprised infrequently forgetting, discontinuing, delaying, or being negligent with their medications.

I just forget it. I don’t have any pain, so until that evening, I’m like, “Wait a minute, did I take my pill?” At that point, I just wait till the next morning. (FG3, Participant 1)

Participants’ reports referred to either past or present nonadherent behaviors. The Figure depicts the emergent factors that determined the position of a participant on the spectrum of adherence. Discontinuing, delaying, or being negligent was typically connected to fear of side effects, a poor patient–physician relationship, and low severity of RA.

**Figure:**
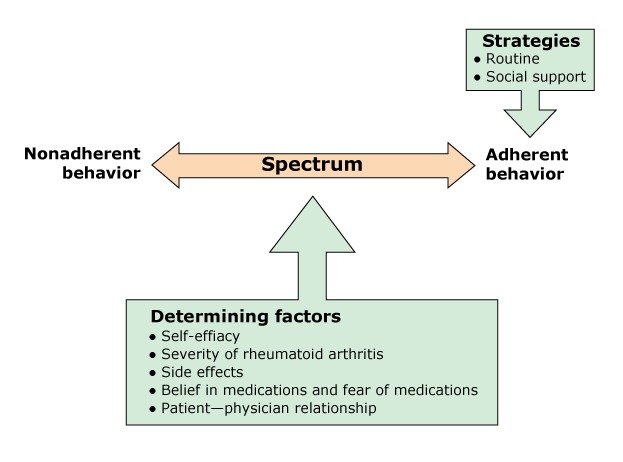
Adherence as a Spectrum of Behaviors. This figure presents the phenomenon of adherence behavior as it relates to medication-taking behavior among rheumatoid arthritis (RA) patients.

I take meloxicam every single day, but a certain point into it, when I start feeling dizzy, I said, ‘No, I’m not taking that anymore.’ (FG2, Participant 5)

These accounts of intermittent nonadherence were often accompanied by expressions of guilt and frustration. They voiced their discomfort with the possible long-term effects of RA medications, and most expressed feeling inconvenienced by taking long-term medications. Nevertheless, most realized the necessity of their medications as pain relievers and mechanisms to stop the progression of RA.

I can definitely say the long-term effects concern me, but right now the benefits outweigh the risks. (FG3, Participant 3)

## Discussion

This study revealed the importance of high self-efficacy in participants’ ability to manage and make adjustments to RA medications. These results are consistent with the few existing studies on the impact of self-efficacy on medication adherence among RA patients (1,19). Most participants reported positive relationships with their providers, and participants who expressed having positive communication with their providers expressed feeling more capable of taking medications appropriately. Self-efficacy was positively influenced by good patient–provider communication, and this implies that the patient–provider relationship could be used as a mechanism to positively influence the self-efficacy of RA patients.

Participants provided perspectives about the role of self-efficacy in enabling them to adjust medications. Individuals with low self-efficacy to adjust medications feared possible side effects and preferred to follow providers’ instructions strictly. Participants with high self-efficacy to adjust medications either did so because of the ineffectiveness of their medications or because they believed they knew their bodies enough to make these adjustments. Accounts of some high self-efficacy participants revealed that some were given leeway from providers to adjust fast-acting medications at their own discretion to manage symptoms.

Patients adjusting prescribed medications is typically classified as nonadherent behavior because this practice does not fit existing definitions of adherence, which consider adherence as a commitment and abidance to the treatment protocol established by the provider (6). But patients with high self-efficacy to adjust medications may better control unpredictable flare-ups and inflammation. This broad classification of adherent versus nonadherent behaviors may complicate the exploration of adherence behaviors in RA patients because the difficulty of managing unpredictable inflammation and flare-ups may be better treated with an adaptable medication regimen. Some rheumatologists employ this circumstantial approach to alleviate pain and flare-ups in patients. This implies that participants who self-adjusted RA medications with permission of their healthcare provider may actually be exhibiting adherent behaviors, as these adjustments are made under the supervision of a healthcare provider.

Participants who stretched time between doses or who increased dosages without alerting their healthcare provider beforehand should be considered nonadherent, as these changes are made without the approval of the provider. But current literature does not differentiate between the aforementioned circumstances of adjusting medications (1–3,19). Thus the classification of all forms of making adjustments to medications as nonadherence might be a misconception of what is actually appropriate medication use by patients who adjust treatments as needed with prior permission from their healthcare provider.

RA adherence research could benefit from adopting the perspective that circumstantial adjustment to medications may be appropriate in cases where immediate access to a healthcare provider is not possible. There is scarce information available about the safe parameters within which adjustments to RA medications can be made (3). Thus, future research should investigate the treatment patterns of rheumatologists in regard to managing variable pain and flare-ups in RA patients, to develop universal pain management protocols to treat RA patients.

The interviews also provided insight into the changes in self-efficacy that participants experienced over time. Some participants disclosed having severe denial of RA when initially diagnosed, and because of this some decided not to adhere to their medication regimen. Most participants who did not experience changes in self-efficacy expressed making efforts to develop and maintain discipline in taking medications. Subsequent research should explore the factors that engender denial of RA. Interventions such as Adherence-Coping Education (ACE) therapy are cost-effective methods of targeting denial (21,22).

Participants depicted adherence to medications as existing on a spectrum of adherent to nonadherent. Many participants revealed moving along the spectrum at any given time as a result of several determining factors and successful implementation of various strategies to facilitate adherent behaviors. Existing literature explores and measures adherence levels as a single outcome; consequently, the examination and perception of adherence as a variable behavior is lacking (2,4). Therefore, these accounts of participants’ adherence behavior as dynamic and variable have significant implications in shifting how patients are viewed in clinical settings. Instead of viewing adherence as dichotomous, adherence should be viewed as variable — patients have the potential to oscillate back and forth between adherent and nonadherent behaviors over time. These findings are supported by some existing literature endeavoring to shift understanding of adherence. For instance, the ABC taxonomy represents adherence as a process comprising 3 phases: initiation, persistence, and discontinuation (4,5).

This study has limitations. It cannot predict the impact of self-efficacy on medication adherence. Participants are from 1 region in North Carolina. To mitigate potential bias during data analysis, research analysts employed an iterative process of analyzing the data independently of one another and reconciling conflicting interpretations of the data through a mediator. 

This study adds valuable context about the adherence behavior of RA patients. The importance of self-efficacy in the ability to manage and adjust medications emerged as a key finding. This finding implies that interventions should be developed and implemented for RA patients with low self-efficacy.
